# Microbiological Assessment of the FilmArray Blood Culture Identification 2 Panel: Potential Impact in Critically Ill Patients

**DOI:** 10.3390/antibiotics12081247

**Published:** 2023-07-29

**Authors:** Carola Mauri, Alessandra Consonni, Elena Briozzo, Chiara Giubbi, Elisa Meroni, Silvia Tonolo, Francesco Luzzaro

**Affiliations:** Clinical Microbiology and Virology Unit, “A. Manzoni” Hospital, 23900 Lecco, Italy

**Keywords:** blood culture, antimicrobial resistance, FilmArray, bloodstream infections, critically ill patients

## Abstract

Rapid pathogen detection and characterization from positive blood cultures are crucial in the management of patients with bloodstream infections (BSI) and in achieving their improved outcomes. In this context, the FilmArray Blood Culture Identification (BCID2) panel is an FDA approved molecular test, which can quickly identify different species and resistance determinants, thus making an impact in antimicrobial practice. In this study, we analyzed 136 positive blood cultures collected from septic critically ill patients from April 2021 to March 2023 by using the FilmArray BCID2 panel, and results obtained by fast molecular analysis were compared to those obtained by routine protocols. Overall, the BCID2 panel showed a strong concordance with conventional methods, particularly in the case of monomicrobial samples, whereas some discrepancies were found in 10/32 polymicrobial samples. Of note, this technique allowed us to identify a significant number of yeasts (37/94 samples) and to unravel the presence of several resistance markers, including both Gram-positive and Gram-negative organisms. These findings strongly support the potential use of the BCID2 panel as an adjunct to the conventional microbiology methods for the management of critically ill septic patients, thus accelerating blood pathogen and resistance genes identification, focusing antibiotic therapy, and avoiding inappropriate and excessive use of drugs.

## 1. Introduction

Bloodstream infections (BSI) are frequent events in critically ill populations and are still strictly related to high mortality and morbidity [1,2]. Fast diagnosis of the causative organism and appropriate antimicrobial therapy are essential for rapid treatment decisions. In this context, the Surviving Sepsis Campaign Guidelines updated in 2021 recommend the administration of appropriate empiric therapy within one hour of making a diagnosis of sepsis or of a patient in shock, with blood cultures being possibly obtained prior to the administration of the antimicrobial agents in order to optimize the timing of pathogen identification [3]. While rapid treatment should be necessary to improve patient outcome, on the other hand, the increasing prevalence of multidrug-resistant (MDR) organisms represents one of the greatest challenges to the effectiveness of BSI treatments [4]. In this perspective, fast microbiology techniques could have an important impact in preventing delays in starting appropriate treatment, reducing unnecessary antibiotic therapies, and minimizing the risk of the selection of MDR strains [5]. Recently, new molecular rapid approaches have been applied in the routine workflow to provide quick pathogen identification and to identify the presence of some resistance genes or resistance profiles [6]. Multiplex PCR platforms are available for this purpose, and in the context of BSI, the BioFire FilmArray Blood Culture ID (BCID) has been proved to be a powerful tool. BCID is a highly multiplexed, single-pouch PCR kit able to identify 24 different pathogens and to give additional insights into some important resistance profiles directly from positive blood cultures [7]. In the last years, an update to the original BCID panel has been released to cover more pathogenic targets linked to BSI and to improve the gene expression analysis of different resistance determinants. In detail, the new BioFire FilmArray BCID2 panel analyzes a total of 43 targets associated with BSI, including 15 Gram-negative bacteria, 11 Gram-positive bacteria, 7 yeast species, and 10 antimicrobial resistance genes including those encoding carbapenemases (IMP, KPC, OXA-48-like, NDM, and VIM), and CTX-M-type extended-spectrum β-lactamases (ESBLs), as well as those responsible for colistin resistance (*mcr-1*), methicillin resistance (*mecA/C,* and specifically for methicillin-resistant *Staphylococcus aureus*—MRSA, *mecA/C* and MREJ—*mec* right-extremity junction) and vancomycin resistance (*vanA/B*) [8]. Unlike BCID, only a few published studies are available in the literature to validate and support BCID2 panel potential functioning and implementation in BSI routine workflows [9,10,11,12,13,14,15,16,17].

In this context, the aim of this study was to evaluate the performance of the molecular BCID2 platform as compared to conventional routine microbiology methods and to assess its possible role as an important value for the management of critically ill septic patients.

## 2. Results

From April 2021 to March 2023, 136 positive blood cultures obtained from critically ill septic patients were studied using the FilmArray BCID2 panel. Of these, ten samples were found negative using FilmArray because the target was not present in the panel (*Saccharomyces cerevisiae*, n = 3; *Acinetobacter radioresistens*, n = 2; *Bacteroides thetaiotaomicron*, n = 1; *Capnocytophaga canimorsus*, n = 1; *Candida guilliermondii*, n = 1, *Eggerthella lenta*, n = 1; *Pasteurella multocida*, n = 1), and then excluded from the study. Therefore, a total of 126 isolates have been analyzed.

### 2.1. Strain Identification

Results obtained by BCID2 panel fully agreed with those observed with routine culture protocol for monomicrobial samples by both rapid and conventional tests in 94 cases (Table 1).

Looking at the group of polymicrobial samples, results obtained by the FilmArray method agreed with the routine culture method in 22 cases (Table 2), while some discrepancies were obtained in 10 samples (Table 3). In particular, BCID2 identified thirty-six microorganisms at the species level and six at the genus level. Concerning samples with discrepancies, the BCID2 panel was unable to identify only one *S. maltophilia*, although five organisms were species off-panel. Otherwise, BCID2 identified eight microorganisms that were not detected by traditional culture methods.

### 2.2. Detection of Resistance

Antimicrobial resistance genes, mainly represented by *bla*CTX-M (n = 16), were detected in 28 samples (Table 4). Among Gram-negatives (n = 59) few carbapenemases were found (*bla*KPC, n = 3). In the Gram-positive (n = 62) group, the following genes were identified: mecA/C in seven *S. epidermidis*, vanA/B in six *E. faecium,* and one mecA/C and MREJ in *S. aureus*. Of note, one sample was simultaneously positive for *E. faecium* and *K. pneumoniae* group revealing both *van*A/B and *bla*KPC genes.

### 2.3. Antimicrobial Therapy

Concerning therapeutic impact, results obtained by the BCID2 panel were evaluated taking into account the ongoing empirical treatment. Use of the rapid molecular method was crucial in 94 cases, thus determining a substantial change compared to an inadequate antimicrobial therapy or providing useful information for setting targeted therapy. In three cases, results obtained by the BCID2 panel were useful for deciding to remove the vascular catheter. In twenty-seven cases, the ongoing antimicrobial treatment was adequate, while in the remaining two cases, the therapy was adjusted based on clinical evolution. Table 5 shows therapy empirically administered to critically ill patients. The impact of the BCID2 panel results on therapeutic choice is reported in Table 6.

## 3. Discussion

Rapid identification of microorganisms responsible for BSI and associated resistance markers are crucial for early initiation of effective antibiotics, thus improving patient outcome, reduced mortality, and appropriate usage of antibiotics [9].

The FilmArray BCID2 panel that recently replaced the BCID panel allows for a commercial molecular approach very complete to this purpose, since 43 targets (of which 10 are resistance determinants) are investigated in the new panel.

According to previous papers [9,12], our study confirms the excellent performance of the BCID2 panel in identifying pathogens causing bloodstream infections and detecting several genes responsible for antimicrobial resistance. Of note, considering monomicrobial samples, only ten pathogens responsible for BSI in critically ill patients (10/104, 9.6%) fell outside the setting of the panel.

Overall, the BCID2 panel showed very good concordance with conventional methods for on-panel organisms, particularly in monomicrobial samples (100% correct identification at genus level, 95.7% correct identification at species level). To this regard, a significant number of yeasts were detected (37/94 samples, taking into account only monomicrobial blood cultures). In particular, a rapid and reliable *Candida* species identification is crucial in bloodstream infections in order to start an effective and adequate antifungal treatment, whereas detection of fungal pathogens by conventional methods is time-consuming [18]. It is notable that based on data from the literature, *Candida* species are an important cause of hospital-acquired bloodstream infections (BSIs) and are associated with the highest mortality rates in healthcare settings [19]. While Candida albicans is still considered the most common species causing BSIs, increasing rates of candidemia due to Candida non-albicans species have been reported [20,21,22].

As also declared by the manufacturer, the panel shows some problems when required to correctly identify BSI pathogens in the case of polymicrobial samples. In our experience, discrepancies were found in 10/32 polymicrobial blood cultures, thus representing a limitation of the panel. Although in five samples, cultural methods revealed microorganisms not included in the panel (i.e., *P. oleovorans*, *C. gilardii*, *E. hirae*). In three cases, *S. epidermidis* was not detected by cultural methods (the gold standard), thus reflecting a possible contamination or the presence of non-vital microorganisms.

Data from the literature reported similar performances of BCID2 in the case of polymicrobial samples. For example, Berinson et al. showed discordances in 12 of 31 polymicrobial samples (38.7%) [9]. In another study, Sparks et al. analyzed fifty-one positive blood cultures, with seven of them showing polymicrobial growth, and there were only two cases where the BCID2 results agreed with those obtained from the reference method [11]. Similarly, Caméléna et al. found discordances in 6 of 22 polymicrobial samples (27.3%) [17]. Only one study (that retrospectively analyzed stored frozen blood culture specimens, including polymicrobial population) found a 100% species identification rate also in those kinds of samples [8]. This limitation of the BCID2 system, regarding the analysis of polymicrobial cultures, is a finding that, to various extents, was previously reported also for the BCID assay [23,24,25].

From a clinical point of view, we demonstrated that using the BCID2 panel concurrently to direct microscopic examination for positive blood cultures had a significant impact for the timely administration of the appropriate antibiotic therapy.

A strong advantage of the system is the ability to detect resistance markers for both Gram-positive and Gram-negative organisms. In Gram-positive organisms, it is notable that the BCID2 panel (differently from the BCID) is able to discriminate between *E. faecalis* and *E. faecium*, as well as to detect vancomycin resistance caused by *van*A/B genes, whose spread is becoming critical in several countries, such as Italy. As already described in the literature [9], this peculiar property could be crucial in the antimicrobial stewardship interventions, thus possibly eventually shortening empirical vancomycin therapies in *Enterococcus* spp. infections, as well as preventing potential escalation in VRE BSI. Indeed, 6 out of 14 *E. faecium* recovered in our study were resistant to vancomycin.

Regarding Gram-negative bacteria, the most common determinants of the third-generation cephalosporin and carbapenem resistance in *Enterobacterales* are covered by the assay. In our study, in detail, resistance markers were found in 16 of 50 samples containing Gram-negative bacteria. However, given the multifactorial molecular basis of cephalosporin and carbapenem resistance in Gram-negative organisms, limitations of molecular assay in predicting susceptibilities against β-lactams are clear. About this, the BCID2 system was unable to detect three microorganisms that most presumably produced AmpC-type enzymes (*K. aerogenes*, n = 2; *E. cloacae*, n = 1) and three bacteria resistant to carbapenems (*P. aeruginosa*, n = 2; *K. aerogenes*, n = 1). However, in these organisms, resistance is often caused by other different mechanisms, not included in the panel. In fact, several *Enterobacterales* and some other Gram-negative bacilli produce natural AmpCs, either constitutively at a trace level or inducibly (i.e., *Enterobacter* spp., *C. freundii*, *M. morganii*, *P. aeruginosa*). The derepression or hyperproduction of natural AmpCs is due to various genetic changes and confers high-level resistance to cephalosporins and to penicillin-β-lactamase inhibitor combinations. Regarding *P. aeruginosa*, this species may be carbapenem resistant through multiple chromosomal mechanisms (active efflux, porin alteration or deficiencies). These resistances were confirmed through phenotypic methods.

Concerning the impact in therapeutical decisional processes, we demonstrated that the application of the rapid molecular method was decisive in removing an inadequate antimicrobial therapy or providing useful information for setting a more targeted therapy. Moreover, our results suggest that the BCID2 panel could also be useful in the rapid identification of infections related to vascular catheters, thus allowing for the quick remotion of these contaminated devices.

Despite this, mainly because of the high costs, molecular approaches such as the BCID2 panel should not be applicable to all patients with suspected BSI. We decided within our study to analyze selected patients in which the assessment of clinical severity and the risk stratification for MDR infection may represent rational inclusive criteria. Future research directions may also be highlighted more in general in clinical use to evaluate clinical outcomes, reduction of unnecessary broad-spectrum antibiotics, and cost-effectiveness.

There are some limitations to the present study. First, this study is focused on critically ill patients; therefore, the potential clinical impact could be influenced. Second, clinical outcomes were not evaluated. However, we analyzed a significant number of yeasts (44/126 blood cultures) while previous studies did not evaluate [14] or investigated only a small number of them [8,9,10,11,12,17].

In conclusion, this study demonstrated that the BCID2 panel has the potential for early and accurate diagnosis of the causative pathogens of BSI, and this molecular platform in combination with routine microbiology methods can improve the contribution for the selection of appropriate antimicrobial agents. Further studies could evaluate the clinical outcomes, reduction of unnecessary broad-spectrum antibiotics, and cost-effectiveness.

## 4. Materials and Methods

Study setting and Inclusion Criteria. This prospective single-center study was conducted from April 2021 to March 2023 at the ASST of Lecco (Italy) that consists of two hospitals for a total of 950 beds. Positive blood culture samples were included in the study if they were collected from critically ill patients. Samples positive for yeast were also investigated. In total, 136 positive blood cultures were studied using both the FilmArray BCID2 panel and the conventional microbiology methods.

**Figure 1:**
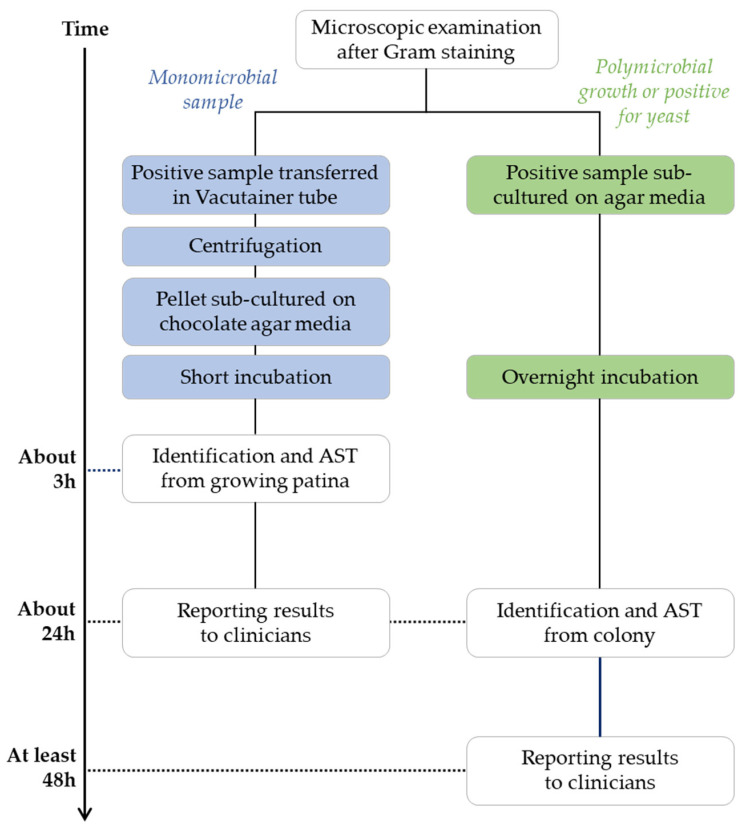
Summary of blood culture diagnostics following conventional culture methods.

**Blood culture diagnostics following conventional culture methods.** Blood culture bottles (BacT/Alert Plus aerobic/anaerobic/pediatric samples, bioMérieux, Marcy l’Etoile, France) were incubated in BacT/AlertVirtuo^®^ instrument (bioMérieux, Marcy l’Etoile, France) at 37 °C. Bottles flagged positive were removed and a Gram stain was performed. From all positive blood cultures, one drop was straked onto different agar plates, including Columbia agar with 5% sheep blood, Chocolate agar with polyvitex, Columbia ANC agar with 5% sheep blood, MacConkey agar, Schaedler agar with Vitamin K1 and 5% sheep blood, and Sabouraud agar with gentamicin and chloramphenicol. Incubation was carried out at 36 °C for a maximum of 48 h in O_2_, 5% CO_2_, or anaerobic atmosphere to enable bacterial growth. If a monomicrobial bacterial population was detected by microscopic examination, an aliquot (2.5 mL) of each sample was transferred in a tube with a gel separator (BD Vacutainer^®^ Blood Collection Tubes, Becton Dickinson and Company, Franklin Lakes, USA) and centrifuged at 3500 rpm for 10 min. The supernatant was discarded and the pellet was inoculated on Chocolate agar with polyvitex plate and incubated at 36 °C in 5% CO_2_ for 3 h. Microbial identification was obtained by MALDI-TOF MS (Vitek MS, bioMérieux) after 3 h or overnight growth. All colonies with different morphotypes were identified on plates after overnight incubation. Susceptibility tests were performed according to the identified microorganism: VITEK 2 instrument (bioMérieux) for non-fastidious bacteria, Kirby-Bauer diffusion susceptibility test according to the EUCAST guidelines for fastidious microorganisms, and Sensititre YeastOne (ThemoFisher Scientific, Waltham, MA, USA) for antifungal sensibility/resistance panel. Results were interpreted according to current EUCAST criteria for bacteria and CLSI criteria for fungi. A flowchart of these conventional diagnostic methods is represented in Figure 1.

**FilmArray BCID2 testing.** FilmArray BCID2 testing was performed according to the manufacturer’s guidelines. Briefly, a hydration solution was loaded into the pouch, and 200 μL of the positive blood culture solution were mixed with the provided sample buffer. This mixture was applied to the pouch. This was subsequently loaded into the instrument. A nucleic acid extraction, a multiplexed nested PCR, and a product melt temperature analysis were performed by the instrument. In only 70 min, this rapid molecular test can detect 43 targets: 33 for the identification of bacteria and fungi, and 10 for the detection of antimicrobial resistance genes (Table 7).

**Impact of the BCID2 panel on antimicrobial therapy.** Organism identification and resistance genes were recorded on a standardized database for both the BCID2 panel and conventional methods. Accuracy of the BCID2 panel was compared to conventional microbiology methods. In detail, for each specimen analyzed, the choice of empirical antimicrobial therapy before communication of the BioFire FilmArray BCID2 panel results, as well as the intended choice of adjusted antimicrobial therapy after the results, were recorded. This information was obtained from the clinician when the BioFire FilmArray BCID2 panel results were relayed.

## Figures and Tables

**Table 1:** Summary of monomicrobial isolates detected by BCID2 panel and then confirmed by routine culture methods. Isolates are grouped on the basis of Gram staining results.

Gram Staining	BCID2 Panel
GPC (n = 19)	*S. aureus* (n = 8) *E. faecium* (n = 7) *E. faecalis* (n = 3) *Staphylococcus* spp. *^a^* (n = 1) *Streptococcus* spp. *^b^* (n = 1)
GPB (n = 3)	*L. monocytogenes* (n = 3)
GNB (n = 35)	*E. coli* (n = 19) *K. pneumoniae* group (n = 8) *P. aeruginosa* (n = 8) *Enterobacterales ^c^* (n = 2) *K. aerogenes* (n = 2) *E. cloacae* complex (n = 1)
Yeast (n = 37)	*C. glabrata* (n = 14) *C. albicans* (n = 10) *C. parapsilosis* (n = 8) *C. tropicalis* (n = 4) *C. neoformans/gattii* (n = 1)

GPC, Gram-positive cocci; GPB, Gram-positive bacilli; GNB, Gram-negative bacilli; ^a^ *Staphylococcus hominis* was identified at genus level as *Staphylococcus* spp.; *^b^ Streptococcus viridans* group was identified at genus level as *Streptococcus* spp.; *^c^ Raoultella ornithinolytica* and *Pantoea* spp. were identified as *Enterobacterales*.

**Table 2:** Summary of concordant microorganism identifications by BCID2 panel and routine culture method in polymicrobial samples.

BCID2 Panel	Routine Culture Method
*C. albicans*, *S. epidermidis*	*C. albicans*, *S. epidermidis*
*C. albicans*, *Staphylococcus* spp.	*C. albicans*, *S. haemolyticus*
*C. glabrata*, *C. tropicalis*, *S. epidermidis*	*C. glabrata*, *C. tropicalis*, *S. epidermidis*
*C. glabrata*, *S. maltophilia*	*C. glabrata*, *S. maltophilia*
*C. tropicalis*, *S. maltophilia*	*C. tropicalis*, *S. maltophilia*
*E. coli*, *E. cloacae* complex	*E. coli*, *E. cloacae* complex
*E. coli*, *E. faecalis*, *E. faecium*	*E. coli*, *E. faecalis*, *E. faecium*
*E. coli*, *K. oxytoca*, *Streptococcus* spp.	*E. coli*, *K. oxytoca*, *S. viridans* group
*E. coli*, *P. aeruginosa*	*E. coli*, *P. aeruginosa*
*E. coli*, *Staphylococcus* spp.	*E. coli*, *S. haemolyticus*
*E. coli*, *S. epidermidis*	*E. coli*, *S. epidermidis*
*E. coli*, *S. epidermidis, Streptococcus* spp.	*E. coli*, *S. epidermidis*, *S. viridans* group
*E. faecalis*, *E. faecium*, *K. aerogenes*	*E. faecalis*, *E. faecium*, *K. aerogenes*
*E. faecalis*, *S. aureus*	*E. faecalis*, *S. aureus*
*E. faecalis, Streptococcus* spp.	*E. faecalis, Streptococcus viridans group*
*E. faecium*, *K. pneumoniae*	*E. faecium*, *K. pneumoniae*
*E. faecium*, *S. aureus*	*E. faecium*, *S. aureus*
*E. faecium, S. epidermidis, Streptococcus* spp.	*E. faecium, S. epidermidis, S. viridans group*
*S. epidermidis*, *Streptococcus* spp. (n = 3)	*S. epidermidis*, *S. viridans* group (n = 3)
*Staphylococcus* spp., *Streptococcus* spp.	*S. capitis*, *S. viridans* group

**Table 3:** Summary of discordant microorganism identifications by BCID2 panel and routine culture method in polymicrobial samples.

BCID2 Panel	Routine Culture Method
*C. glabrata*, *C. tropicalis*	*C. tropicalis*, *Pseudomonas oleovorans*, *Cupriavidus gilardii*
*C. glabrata*, *C. tropicalis*	*C. glabrata*
*E. faecalis, E. cloacae complex, E. coli, K. oxytoca,* *S. maltophilia*, *Streptococcus* spp.	*E. faecalis*, *E. cloacae*, *E. coli*, *K. oxytoca*
*E. faecalis*, *S. epidermidis*, *Staphylococcus* spp.	*E. faecalis*, *S. haemolyticus*
*E. faecium*, *S. epidermidis*, *Staphylococcus* spp.	*E. faecium*, *S. hominis*
*E. faecium*, *S. epidermidis*	*E. faecium*
*E. coli*, *K. oxytoca*	*E. coli*, *E. hirae*, *K. oxytoca*
*E. coli*, *K. pneumoniae group*, *P. aeruginosa*	*Aeromonas caviae*, *K. pneumoniae*, *P. aeruginosa*
*P. aeruginosa, S. epidermidis*	*Achromobacter xylosoxidans*, *P. aeruginosa*, *S. epidermidis*
*S. pneumoniae*	*S. pneumoniae*, *S. maltophilia*

**Table 4:** Samples with resistance genes detected by BCID2 panel and results from routine culture method.

N	BCID2 Panel	Routine Culture Method
6	*E. coli,* CTX-M	ESBL-producing *E. coli*
3	*K. pneumoniae* group, CTX-M	ESBL-producing *K. pneumoniae*
3	*E. faecium*, *van*A/B	Vancomycin-resistant *E. faecium*
2	*K. pneumoniae* group, CTX-M, KPC	KPC-producing *K. pneumoniae*
2	*S. epidermidis, Streptococcus* spp., *mec*A/C	Methicillin resistant *S. epidermidis*, S. viridans group
1	*C. albicans*, *S. epidermidis*, *mec*A/C	*C. albicans*, methicillin-resistant *S. epidermidis*
1	*E. cloacae complex, E. coli*, CTX-M	*E. cloacae complex*, ESBL-producing *E. coli*
1	*E. cloacae complex, E. coli*, *E. faecalis*, *K. oxytoca*, *S. maltophilia*, *Streptococcus* spp., CTX-M	*E. cloacae complex,* ESBL-producing *E. coli*, *E. faecalis*, *K. oxytoca*
1	*E. coli*, *E. faecalis*, *E. faecium*, CTX-M	ESBL-producing *E. coli*, *E. faecalis*, *E. faecium*
1	*E. coli*, *S. epidermidis,* CTX-M, *mec*A/C	ESBL-producing *E. coli*, methicillin-resistant *S. epidermidis*
1	*E. coli*, *S. epidermidis, Streptococcus* spp., CTX-M	ESBL-producing *E. coli*, *S. epidermidis*, *S. viridans* group
1	*E. faecium*, *K. pneumoniae, van*A/B, KPC	Vancomycin-resistant *E. faecium*, KPC-producing *K. pneumoniae*
1	*E. faecium*, *S. aureus, van*A/B	Vancomycin-resistant *E. faecium*, *S. aureus*
1	*E. faecium*, *S. epidermidis*, *mec*A/C	*E. faecium*
1	*E. faecium*, *S. epidermidis, Staphylococcus* spp., *mec*A/C, *van*A/B	Vancomycin-resistant *E. faecium*, methicillin-resistant *S. hominis*
1	*E. faecium*, *S. epidermidis, Streptococcus* spp., *mec*A/C	*E. faecium*, methicillin-resistant *S. epidermidis*, *S. viridans* group
1	*S. aureus mec*A/C and MREJ	Methicillin-resistant *S. aureus*

**Table 5:** Summary of empiric therapy and adequacy in relation to microbiological results.

Empiric Therapy	Total.	No Therapy Change	Therapy Set or Adjusted	Other
Antimicrobial combination therapy	36	7	29	-
β-lactam agent + inhibitor	27	8	19	-
Therapeutic window	26	1	22	3
Third generation cephalosporin	19	6	13	-
Carbapenem (meropenem)	7	2	5	-
Antifungal agent	6	2	3	1
Glicopeptide (vancomycin)	4	1	2	1
Penicillin (oxacillin)	1	-	1	-
Total	126	27	94	5

**Table 6:** Impact of BCID2 panel results on therapeutic choice.

BCID2 Result	No Therapy Change	Set Up Targeted Therapy	Therapy Changed or Optimized		Other
			Organism Based	Gene Based	
Staphylococci (n = 8)	3	-	4	1	-
Streptococci and Enterococci (n = 11)	2	1	5	2	1
*L. monocytogenes* (n = 3)	-	-	3	-	-
*Enterobacterales* (n = 32)	13	8	3	7	1
*P. aeruginosa* (n = 3)	1	1	1	-	-
Yeast (n = 37)	1	7	28	-	1
Polymicrobial with bacteria (n = 25)	6	2	9	6	2
Polymicrobial with yeast (n = 7)	1	3	3	-	-
Total	27	22	56	16	5

**Table 7:** Summary of targets available for testing on the BioFire FilmArray BCID2 panel.

**Gram-Positive Bacteria**	**Gram-Negative Bacteria**
*Enterococcus faecalis* *Enterococcus faecium* *Listeria monocytogenes* *Staphylococcus* *Staphylococcus aureus* *Staphylococcus epidermidis* *Staphylococcus lugdunensis* *Streptococcus* *Streptococcus agalactiae* *Streptococcus pyogenes* *Streptococcus pneumoniae*	*Acinetobacter calcoaceticus-baumannii complex* *Bacteroides fragilis* *Enterobacterales* *Enterobacter cloacae complex* *Escherichia coli* *Klebsiella aerogenes* *Klebsiella oxytoca* *Klebsiella pneumoniae group* *Proteus* *Salmonella* *Serratia marcescens* *Haemophilus influenzae* *Neisseria meningitidis* *Pseudomonas aeruginosa* *Stenotrophomonas maltophilia*
**Yeast**	**Antibiotic Resistance**
*Candida albicans* *Candida auris* *Candida glabrata* *Candida krusei* *Candida parapsilosis* *Candida tropicalis* *Cryptococcus neoformans/gattii*	Carbapenemases: IMP, KPC, OXA-48-like, NDM, VIM Colistin Resistance: *mcr*-1 ESBL: CTX-M Methicillin Resistance: *mec*A/C, *mec*A/C and MREJ (MRSA) Vancomycin Resistance: *van*A/B

## Data Availability

All data relevant to this study are included in the article.
